# Physiologic medium renders human iPSC-derived macrophages permissive for *M. tuberculosis* by rewiring organelle function and metabolism

**DOI:** 10.1128/mbio.00353-24

**Published:** 2024-07-10

**Authors:** Claudio Bussi, Rachel Lai, Natalia Athanasiadi, Maximiliano G. Gutierrez

**Affiliations:** 1 The Francis Crick Institute, London, United Kingdom; 2 School of Biological Sciences, Nanyang Technological University, Singapore, Singapore; 3 Department of Infectious Diseases, Imperial College London, London, United Kingdom; Leibniz-Institut fur Naturstoff-Forschung und Infektionsbiologie eV Hans-Knoll-Institut, Jena, Germany; Research Center Borstel, Borstel, Germany

**Keywords:** macrophages, *Mycobacterium tuberculosis*, metabolism, organelle, physiologic media, RNA-Seq, lysosomes, lipid droplets, mitochondria

## Abstract

*In vitro* studies are crucial for our understanding of the human macrophage immune functions. However, traditional *in vitro* culture media poorly reflect the metabolic composition of blood, potentially affecting the outcomes of these studies. Here, we analyzed the impact of a physiological medium on human induced pluripotent stem cell (iPSC)-derived macrophages (iPSDM) function. Macrophages cultured in a human plasma-like medium (HPLM) were more permissive to *Mycobacterium tuberculosis* (Mtb) replication and showed decreased lipid metabolism with increased metabolic polarization. Functionally, we discovered that HPLM-differentiated macrophages showed different metabolic organelle content and activity. Specifically, HPLM-differentiated macrophages displayed reduced lipid droplet and peroxisome content, increased lysosomal proteolytic activity, and increased mitochondrial activity and dynamics. Inhibiting or inducing lipid droplet formation revealed that lipid droplet content is a key factor influencing macrophage permissiveness to Mtb. These findings underscore the importance of using physiologically relevant media *in vitro* for accurately studying human macrophage function.

**IMPORTANCE:**

This work compellingly demonstrates that the choice of culture medium significantly influences *M. tuberculosis* replication outcomes, thus emphasizing the importance of employing physiologically relevant media for accurate in vitro host-pathogen interaction studies. We anticipate that our work will set a precedent for future research with clinical relevance, particularly in evaluating antibiotic efficacy and resistance *in cellulo*.

## INTRODUCTION

Human macrophage research has largely relied on macrophages differentiated from monocytes *in vitro*, an approach that has significantly contributed to our understanding of macrophage biology. Recent advancements in generating macrophages from induced pluripotent stem cells (iPSCs) have expanded the repertoire of experimentally accessible systems for studying human macrophage biology (1, 2). Despite these advances, the culture and differentiation of iPSC-derived macrophages (iPSDM), like many cell biology and metabolic studies utilizing various *in vitro* models, employ culture media that poorly represent the metabolite composition of human plasma (1, 3–5). Consequently, the influence of the small molecule composition of culture media on macrophage metabolism and cellular function remains largely unexplored.

Traditional cell culture media, such as DMEM and RPMI 1640, are formulated with glucose, amino acids, vitamins, and salts at concentrations that significantly differ from those found in human plasma. Additionally, these media are missing various components that have been identified in plasma through mass spectrometry and nuclear magnetic resonance (NMR) spectroscopy analysis (3, 5, 6). Typically, these basal media are enriched with fetal bovine serum (FBS), which introduces a complex and often overlooked mixture of polar metabolites and lipids, alongside essential growth factors and hormones necessary for cell growth, which may differ depending on the lot and origin of the serum (7, 8).

Over recent years, there have been numerous initiatives aimed at modifying cell culture media compositions to more closely mimic physiological conditions. These initiatives have led to the development of two media that mirror the metabolic composition of human plasma, specifically, human plasma-like medium (HPLM) and Plasmax (3, 5, 8, 9). These media contain more than 60 polar metabolites such as amino acids, nucleic acids, sugars, and small organic acids at concentrations found in human plasma. To minimize the impact of serum on plasma metabolite composition, the developers of Plasmax medium lowered its serum concentration to 2.5%, whereas the developers of the HPLM medium opted to substitute the typical serum with a dialyzed version devoid of polar metabolites (3, 5).

Adding further complexity, human macrophage differentiation *in vitro*, either from human blood-derived monocytes or iPSC-derived monocytes, typically involves the cytokines macrophage colony-stimulating factor (M-CSF) and granulocyte-macrophage colony-stimulating factor (GM-CSF). M-CSF is continuously produced in most tissues and regulates the population of macrophages in numerous organs. In contrast, GM-CSF is primarily produced in the lungs and typically has low baseline levels in the circulation, which tend to increase in response to immune or inflammatory events (10). M-CSF operates through the c-FMS receptor (CD115), a single-pass transmembrane protein tyrosine kinase that belongs to the PDGF family. Conversely, GM-CSF utilizes CD116, an α/β heterodimeric receptor, the β-chain of which is shared with interleukin-3 (IL-3) and IL-5 (10, 11). Although previous studies have compared the gene expression profiles of human macrophages differentiated with M-CSF and GM-CSF (12), the rationale behind selecting a specific differentiation protocol and the impact of these differentiation programs on macrophage organelle activity and composition remain poorly investigated.

In this study, we address these critical gaps by integrating RNA sequencing, extracellular flux analyses, and high-content single-cell imaging to comprehensively evaluate the effects of culture media composition on human macrophage organelle activity and host cell response to the intracellular pathogen *Mycobacterium tuberculosis* (Mtb). We discovered that macrophages in physiological media displayed distinct proteolytic and lipid droplet content, mitochondrial activity, and metabolic properties with an impact on the ability of these cells to control Mtb. We anticipate that this study will not only contribute to establishing more standardized iPSC-derived cells culture practices but also illuminating the mechanisms by which modulating organelle activity influences macrophage effector functions.

## RESULTS

### HPLM-differentiated macrophages are more permissive for Mtb replication

**Fig 1:**
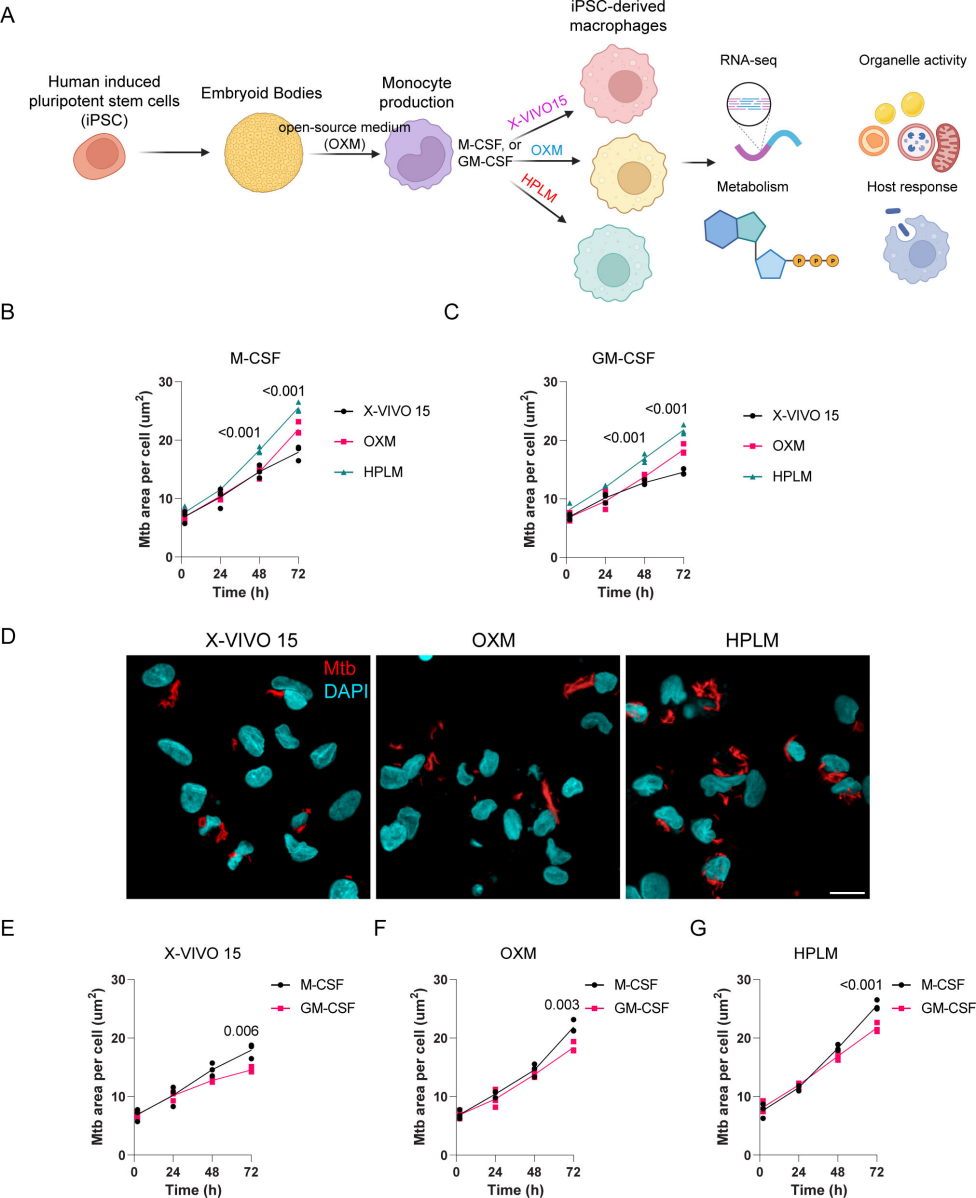
iPSDM cultured in the physiological medium are more permissive to *M. tuberculosis* replication. (A) Schematic illustrating the experimental conditions evaluated. (B, C) Quantification of Mtb replication (bacteria area per cell) in iPSDM differentiated with M-CSF or GM-CSF and cultured with the indicated media, *n* = 3, two-way analysis of variance (ANOVA). (D) Representative images of Mtb-infected iPSDM after 72 h (MOI = 1) and cultured in different media. Scale bar = 20 mm. (E–G) Quantification of Mtb replication (bacteria area per cell) in iPSDM differentiated with M-CSF or GM-CSF and cultured with X-VIVO15, OXM, or HPLM, *n* = 3, two-way ANOVA, Sidak’s multiple comparison test.

Open-source medium (OXM) has been established to culture both induced pluripotent stem cells (iPSC) and induced pluripotent stem cell-derived macrophages (iPSDM). The OXM is a differentiation medium based on Advanced DMEM/F-12 (aDMEM/F-12) that has been developed as an alternative to commercial, serum-free media of undisclosed composition, such as X-VIVO 15 (1, 4). Although the use of OXM offers practical advantages and provides a more standardized method for iPSDM production, its chemical composition still differs significantly from human plasma. For instance, the glucose concentration of OXM is 16.7 mM, approximately 3 to 4 times the concentration found in human plasma (3–5). To overcome this limitation and establish a differentiation method using physiologic medium, we compared and functionally characterized iPSDM, differentiated using M-CSF or GM-CSF in either X-VIVO15, OXM, or Human Plasma-Like Medium (HPLM) (3) while maintaining the iPSC factories for monocyte production using OXM as previously described (4) (Fig. 1A). We first determined by flow cytometry different monocyte and macrophage surface markers to evaluate macrophage phenotypic differences among the different media. We found that the surface marker abundance of CD16, CD14, CD119, CD206, CD163, CD169, and CD86 was similar in macrophages cultured in X-VIVO15, OXM, or HPLM consistent with previous reports (1, 2) (see Fig. S1A and B at https://doi.org/10.25418/crick.26048884).

Given that one of the most relevant functions of macrophages is phagocytosis and bacterial killing (13), we studied the functional outcomes of macrophage differentiation under physiologic and non-physiologic media in infection. We investigated the ability of macrophage populations to restrict the replication of the human pathogen Mtb using single-cell high-content imaging. We observed that Mtb replication in HPLM-differentiated macrophages was greater than with OXM and X-VIVO15 (Fig. 1B through D). In addition, GM-CSF-differentiated macrophages presented less Mtb growth in comparison to M-CSF differentiation across all the media tested (Fig. 1E through G). Overall, our results highlight important consequences for the host cell response to Mtb infection when macrophages are differentiated in HPLM and identify that macrophages growing in a physiologic medium represent a more permissive environment for Mtb replication.

### X-VIVO15- and OXM-differentiated macrophages show a lipid metabolism-associated transcriptional signature

**Fig 2:**
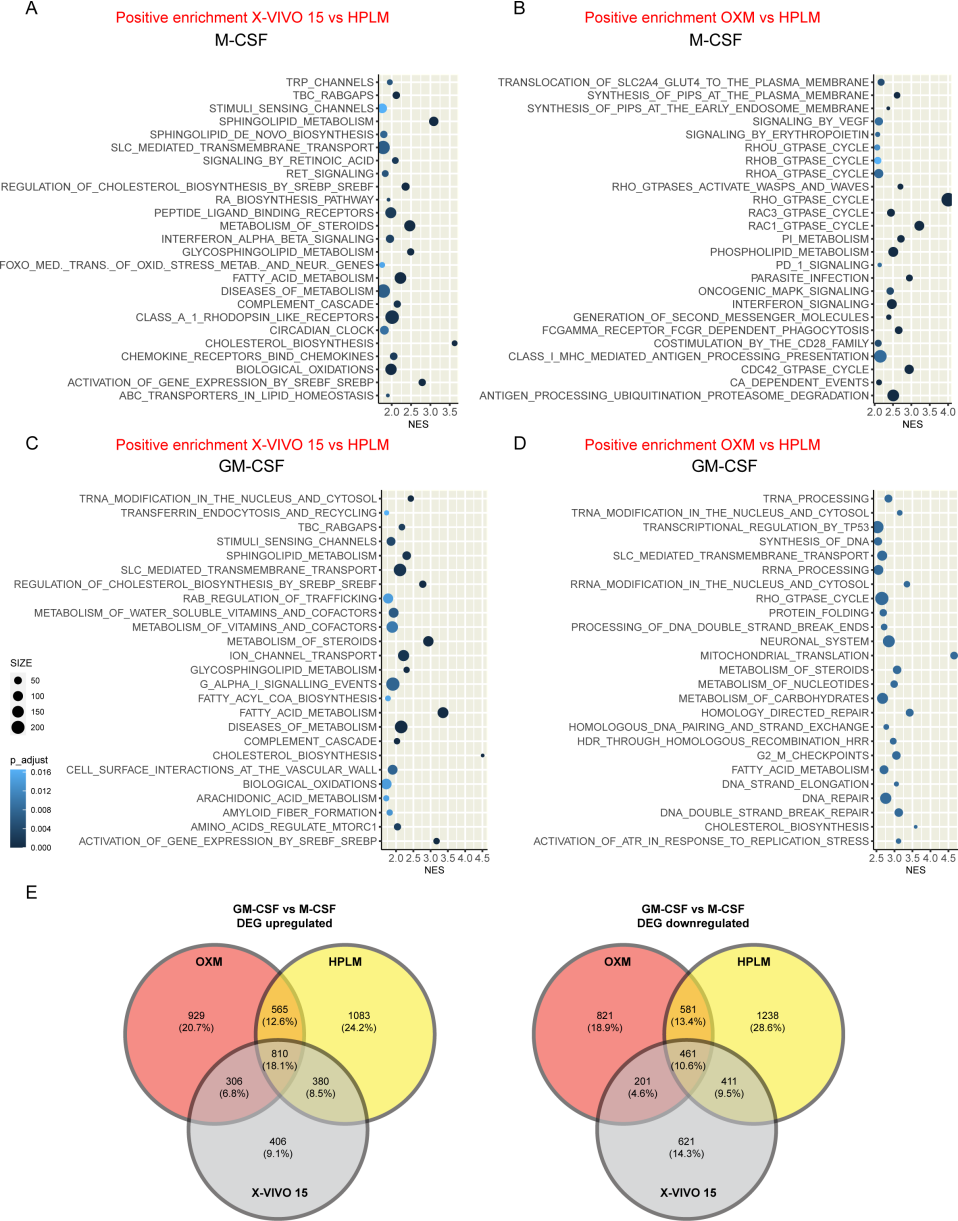
iPSDM cultured in a physiological medium shows a decrease in lipid metabolism-associated transcriptional signature. Top 25 pathways significantly enriched in GSEA (*P*_adj_ < 0.05) ranked by NES (normalized enrichment score). (A, B) Plots show the GSEA results of iPSDM differentiated with M-CSF and cultured with X-VIVO15 or OXM in comparison with HPLM. (C, D) Plots show the GSEA results of iPSDM differentiated with GM-CSF and cultured with X-VIVO15 or OXM in comparison with HPLM. *n* = 3 technical replicates. Note that only the top 25 regulated pathways are plotted; for a comprehensive list of all significantly regulated pathways, please refer to Table S1 at https://doi.org/10.25418/crick.26048884. (E) Venn diagrams show upregulated (left) or downregulated (right) DEGs (filtered by fold change > 1.2 and adjusted *P*-value < 0.05 when comparing GM-CSF with M-CSF) in each culture medium.

To further investigate the phenotypic distinctions between HPLM-, OXM-, and X-VIVO15-differentiated macrophages with either M-CSF or GM-CSF, we employed bulk RNA sequencing (RNA-seq) analysis. Utilizing gene set enrichment analysis (GSEA) with REACTOME, we identified over 100 distinct pathways selectively enriched among the various media tested (Fig. 2; see Fig. S2 and Table S1 at https://doi.org/10.25418/crick.26048884). We found that GM-CSF differentiation induced enrichment in several IFN-associated pathways, including interferon, interferon gamma, and interferon alpha beta signaling, as well as interleukin (IL) pathways such as IL-10 and IL-13, compared with M-CSF-differentiated iPSDM (see Fig. S2 and S3 at https://doi.org/10.25418/crick.26048884). Although these alterations constituted a common transcriptional signature across the three different media tested, both OXM and X-VIVO15 differentiations triggered an enrichment in lipid metabolism-associated programs, such as sphingolipid, phospholipid, cholesterol, and fatty acid metabolism pathways, compared with HPLM-differentiated iPSDM (Fig. 2A through D; see Table S1 at https://doi.org/10.25418/crick.26048884). Notably, this enhanced lipid metabolism-associated transcriptional signature was prevalent for both M-CSF and GM-CSF differentiation programs and more pronounced in X-VIVO15-differentiated HPLM. Consistent with these findings, a previous study demonstrated enrichment in lipid metabolic pathways in X-VIVO15-differentiated iPSDM compared with OXM media (4). However, we observed an unappreciated increase in lipid metabolism that also applies to macrophages differentiated in OXM when compared with HPLM-differentiated iPSDM (Fig. 2A through D; see Tables S1 and S2 at https://doi.org/10.25418/crick.26048884). To further explore the effect of cell culture media on macrophage transcriptional programs, we analyzed the number of genes up- and downregulated when comparing GM-CSF with M-CSF-differentiated macrophages, independent of the media used (Fig. 2E; see Table S3 at https://doi.org/10.25418/crick.26048884). Intriguingly, only 18.1% (810 transcripts) of the differentially expressed genes (DEGs) were commonly upregulated. These genes were mainly associated with interferon signaling pathways. Additionally, a smaller fraction of genes (10.6%) was commonly downregulated, with transcripts associated with rRNA processing and Rho pathways, among others (Fig. 2E; see Table S3). Overall, these results highlight the profound impact of the cell culture media on macrophage transcript signatures.

### Physiologic medium-differentiated macrophages display increased metabolic polarization

**Fig 3:**
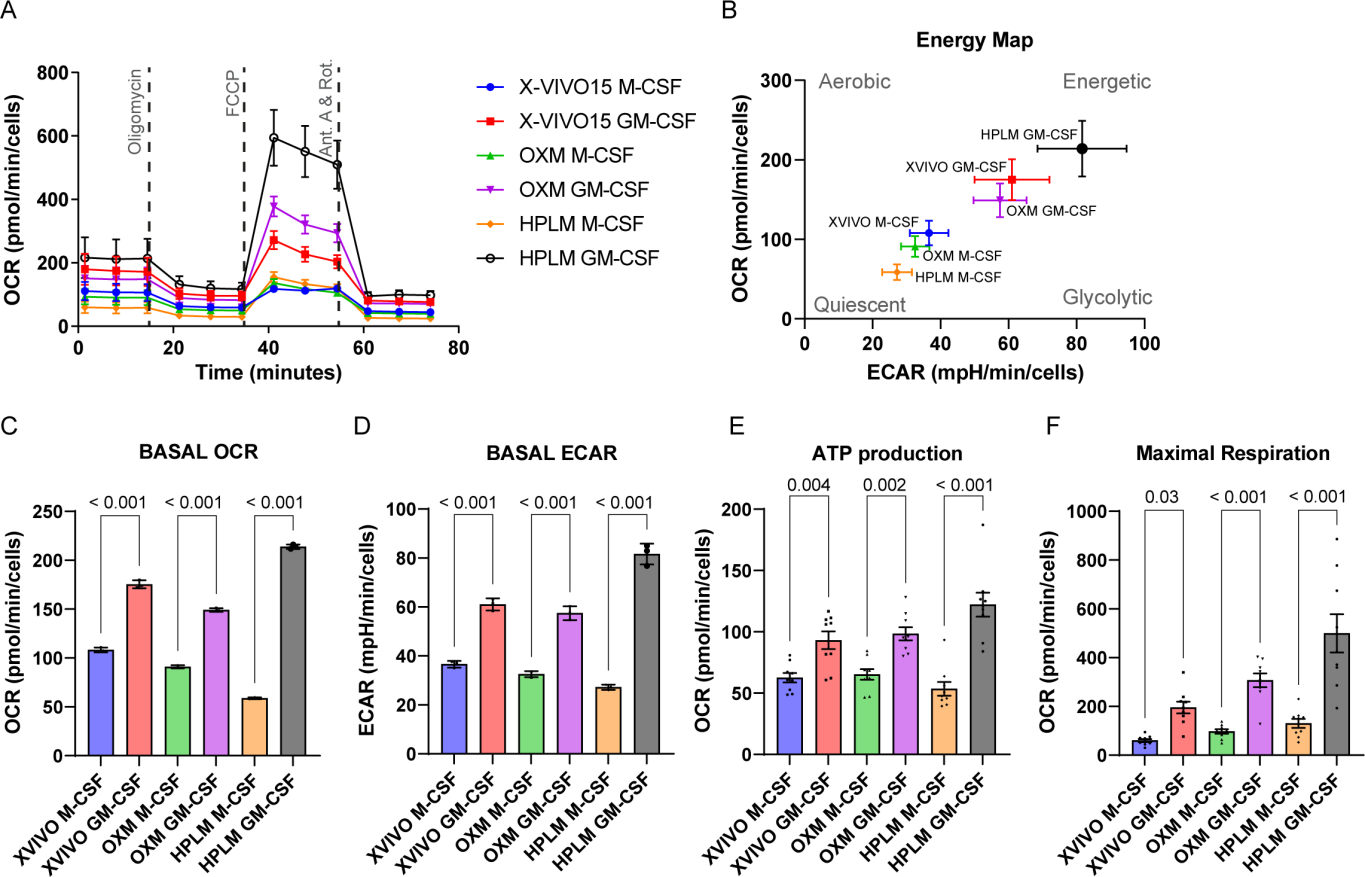
iPSDM cultured in the physiological medium lead to cellular metabolism reprogramming. (**A**) OCR profile at baseline and in response to oligomycin, carbonyl cyanide 4-(trifluoromethoxy) phenylhydrazone (FCCP), and antimycin A plus rotenone (ant. A + Rot) of iPSDM differentiated with M-CSF or GM-CSF and cultured with the indicated media. **(**B) Energy map showing the OCR and extracellular acidification rate (ECAR) basal values of iPSDM cultured and differentiated as before. (C–F) Bar plots show quantification of basal OCR, basal ECAR, ATP production, and maximal respiration of iPSDM cultured and differentiated as in panel **A**. Results are normalized to total cell numbers and show one out of two independent experiments with *n* ≥ 7 technical replicates. *P* values were calculated using a two-tailed *t*-test.

Because our gene expression analysis suggested important changes in the metabolism of HPLM-differentiated macrophages, we next investigated the homeostatic metabolism and the response to mitochondrial activity uncouplers of iPSDM cultured in physiologic and non-physiologic media by extracellular flux analysis (14) (Fig. 3A). Although we observed that HPLM-differentiated macrophages in M-CSF showed the lowest basal oxygen consumption rate (OCR; indicative of OXPHOS), we found that GM-CSF triggered a macrophage metabolic polarization toward an energetic phenotype, characterized by an increase in OCR and extracellular acidification rate (ECAR; indicative of glycolysis) in all the media evaluated (Fig. 3B through D). These results align with previous studies that demonstrated the polarization of GM-CSF-differentiated macrophages toward a glycolytic profile (15, 16).

Moreover, HPLM-differentiated macrophages displayed the greatest increase in OCR and ECAR when compared GM-CSF with M-CSF differentiation (Fig. 3B through D). In line with these results, the response to mitochondrial stressors evaluated through ATP-linked respiration and maximal respiration parameters was also greater for GM-CSF-differentiated macrophages cultured in HPLM, in comparison with OXM and X-VIVO 15 media (Fig. 3A, E, and F). This observation underscores the differential sensitivity of macrophages to their metabolic environment, suggesting that GM-CSF-differentiated macrophages may exhibit a heightened responsiveness to media composition in terms of metabolic activity. Overall, our results argue that medium resembling human plasma composition results in a more extensive metabolic plasticity (17) for human macrophages.

### Physiologic medium-differentiated macrophages show low lipid droplet content and increased lysosomal proteolytic activity

**Fig 4:**
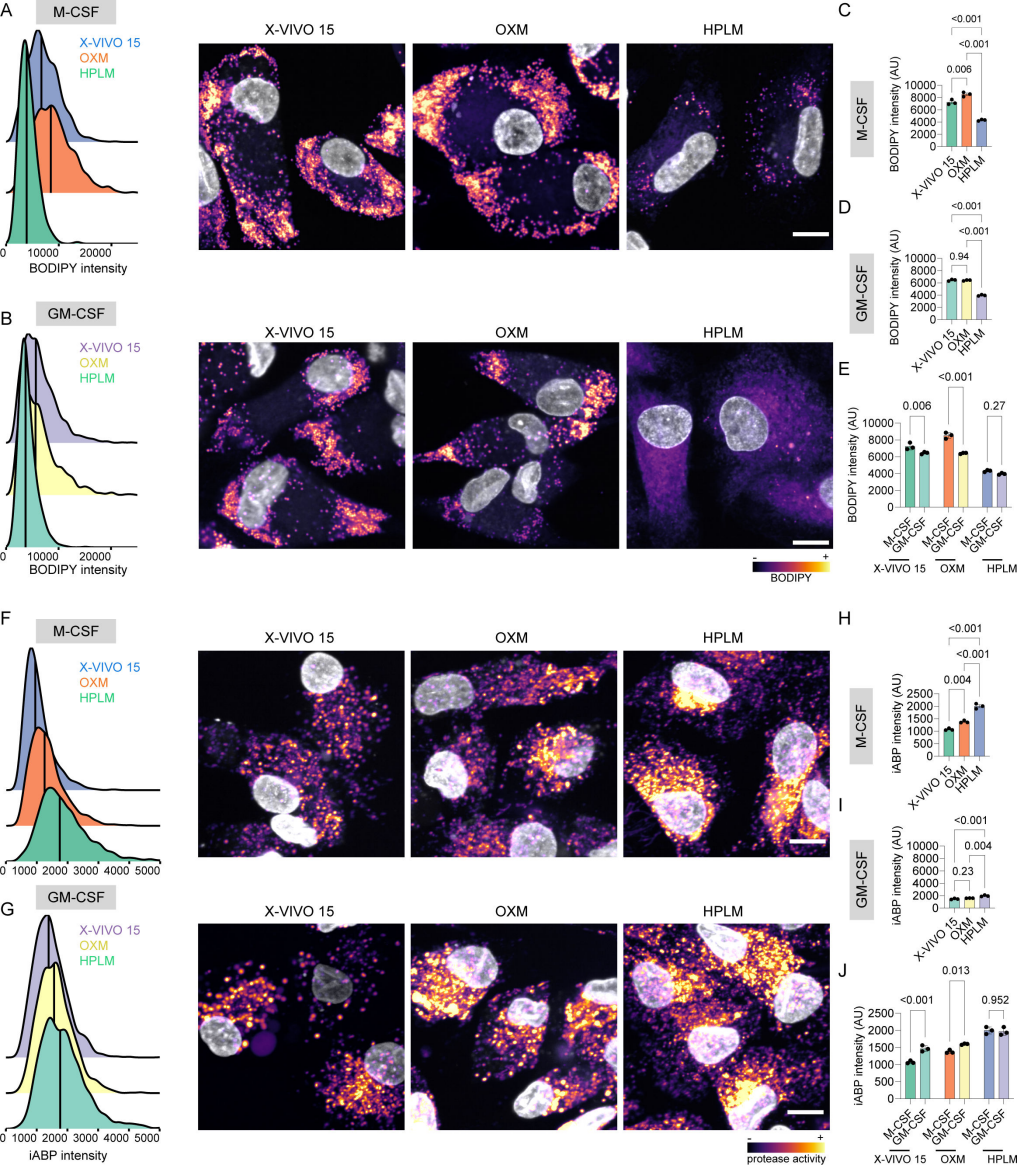
iPSDM cultured in physiological medium changes iPSDM protease activity and lipid droplet content. (A, B) BODIPY intensity density plot (left) and representative images (right) of iPSDM differentiated with M-CSF or GM-CSF and cultured with the indicated media in the presence of BODIPY. (C, D) Bar plots show BODIPY intensity quantification of iPSDM differentiated with M-CSF and GM-CSF and cultured with the indicated media. *P* values were calculated using ANOVA, Tukey post-hoc test. (E) BODIPY intensity quantification showing the effect of M-CSF and GM-CSF differentiation program on iPSDM cultured with the indicated media. *P* values were calculated using a two-way ANOVA, Sidak’s multiple comparison test. (F, G) iABP (lysosomal protease activity-based probe) intensity density plot (left) and representative images (right) of iPSDM differentiated with M-CSF or GM-CSF and cultured with the indicated media in the presence of iABP probe. (H, I) Bar plots show iABP intensity quantification of iPSDM differentiated with M-CSF and GM-CSF and cultured with the indicated media. *P* values were calculated using ANOVA, Tukey post-hoc test. (J) iABP intensity quantification showing the effect of M-CSF and GM-CSF differentiation program on iPSDM cultured with the indicated media. *P* values were calculated using a two-way ANOVA, Sidak’s multiple comparison test. *n* = 3 independent experiments, scale bar: 10 mm.

Macrophage metabolism is recognized as one of the main factors that regulate immune function, and it has been primarily studied *in vitro*. Given that our RNA-seq analysis revealed a selective enrichment in lipid metabolism-associated pathways for macrophages differentiated in X-VIVO 15 and OXM in comparison to HPLM-differentiated iPSDM, we sought to determine how these different transcriptional programs impact iPSDM organelle homeostasis. We first evaluated the macrophage lipid droplet (LD) content by using high-content single-cell imaging and fluorinated boron-dipyrromethene (BODIPY) staining (18). Strikingly, we observed that X-VIVO15-differentiated macrophages showed the highest amount of LD content per cell (evaluated as mean BODIPY intensity and mean droplet area per cell), followed by OXM-differentiated macrophages (Fig. 4A through D; see Fig. S4A and B at https://doi.org/10.25418/crick.26048884). In contrast, HPLM-differentiated macrophages showed very low levels of LD. We also observed a decrease in the LD content in GM-CSF-differentiated macrophages when compared with M-CSF-differentiated macrophages (Fig. 4E). Endolysosomal degradation is central to lipid homeostasis via clearance of extracellular lipoproteins and autophagy (19, 20). Thus, we investigated if the observed differences in LD content were related to differing lysosomal and proteolytic activities. By using a pan-cathepsin activity-based probe (21), we found that HPLM-differentiated macrophages showed a 2-fold increase in proteolytic activity in comparison with OXM- or XVIVO15-differentiated macrophages (Fig. 4F through I). These results were consistent with a marked increase in lysosomal content (evaluated as lysosomal area per cell) observed in HPLM-differentiated macrophages (see Fig. S4C and D at https://doi.org/10.25418/crick.26048884). In addition, we observed that GM-CSF differentiation triggered an increase in lysosomal proteolytic activity in comparison with M-CSF differentiation for OXM-differentiated macrophages or XVIVO15-differentiated macrophages (Fig. 4H through J). Overall, these results show an inverse correlation between lipid droplet content and proteolytic activity for HPLM-differentiated iPSDM, suggesting that media composition is the main factor regulating macrophage organelle content, activity, and function.

### Physiologic medium-differentiated macrophages display increased mitochondrial activity

**Fig 5:**
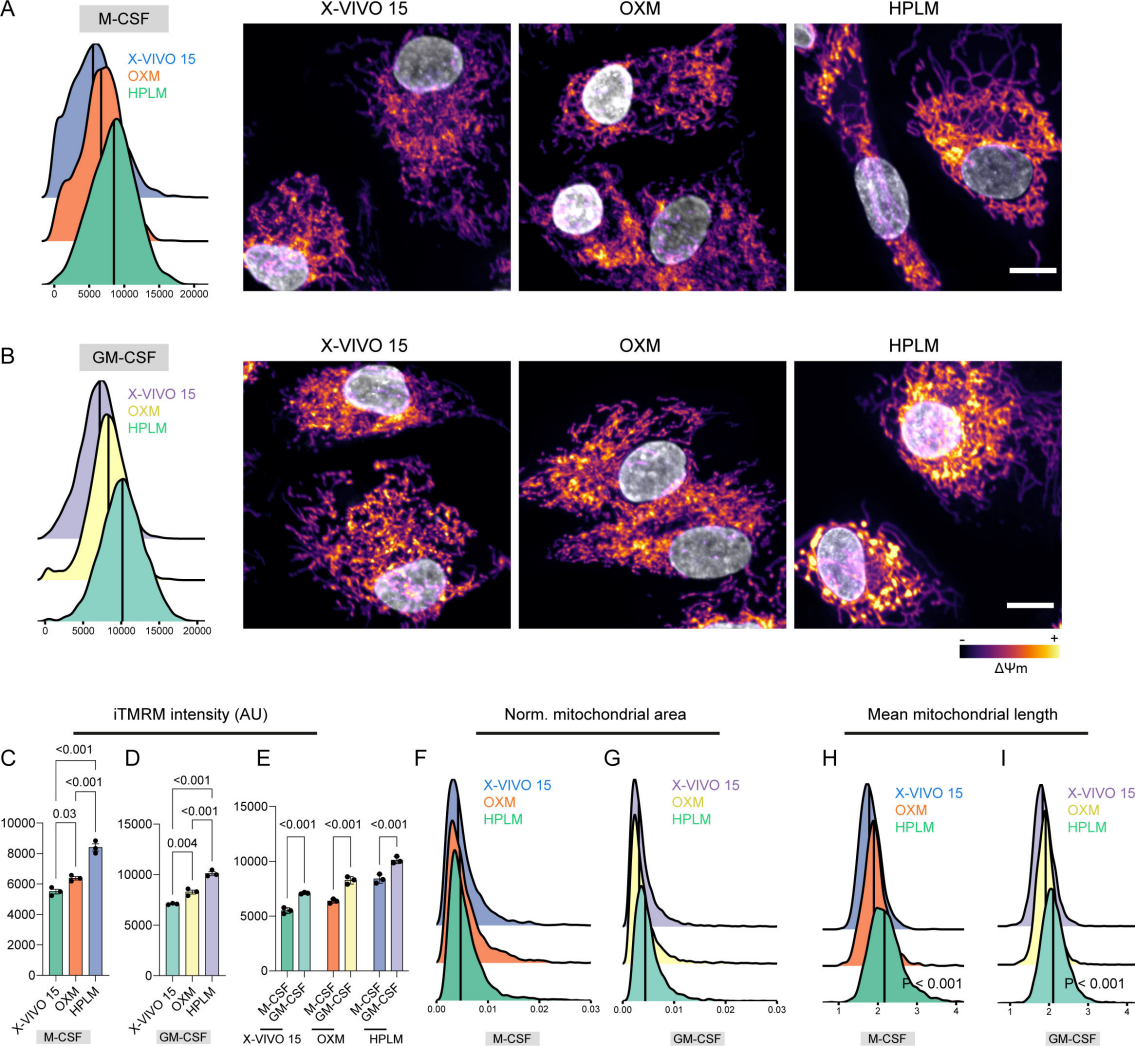
Cell culture media modulates mitochondrial activity. (A, B) iTMRM (mitochondrial activity probe) intensity density plot (left) and representative images (right) of iPSDM differentiated with M-CSF or GM-CSF and cultured with the indicated media in the presence of iTMRM. (C, D) Bar plots show iTMRM intensity quantification of iPSDM differentiated with M-CSF and GM-CSF and cultured with the indicated media. *P* values were calculated using ANOVA, Tukey post-hoc test. (E) iTMRM intensity quantification showing the effect of M-CSF and GM-CSF differentiation program on iPSDM cultured with the indicated media. *P* values were calculated using a two-way ANOVA, Sidak’s multiple comparison test. (F, G) Density plots showing the mitochondrial area values (normalized to cell area) of iPSDM differentiated with M-CSF and GM-CSF and cultured with the indicated media. (H, I) Density plots showing the mean mitochondrial length values of iPSDM differentiated with M-CSF and GM-CSF and cultured with the indicated media. *n* = 3 independent experiments, scale bar: 10 mm.

Lipids constitute a primary mitochondrial source of energy; however, there is evidence suggesting that they can also act as uncouplers and inhibitors of OXPHOS (22). A single-cell mitochondrial segmentation analysis to quantify the mitochondrial accumulation of the membrane-sensitive dye tetramethylrhodamine methyl ester (TMRM) (14) showed that HPLM-differentiated macrophages had increased mitochondrial activity (Fig. 5A through D). Moreover, we observed that GM-CSF differentiation increased the mitochondrial activity of iPSDM cultured in X-VIVO 15, OXM, and HPLM (Fig. 5E). Although we did not observe changes in the mitochondrial mass (quantified as total mitochondrial area per cell) (Fig. 5F and G), we found that HPLM-differentiated macrophages displayed a more elongated mitochondrial network (Fig. 5H and I). Altogether, these results confirm an increased metabolic reprogramming ability observed in HPLM-differentiated macrophages by extracellular flux analysis and indicate that physiologic media triggers an increase in mitochondrial activity.

### Physiologic medium-differentiated macrophages display increased mitochondrial dynamics and different peroxisome contents

**Fig 6:**
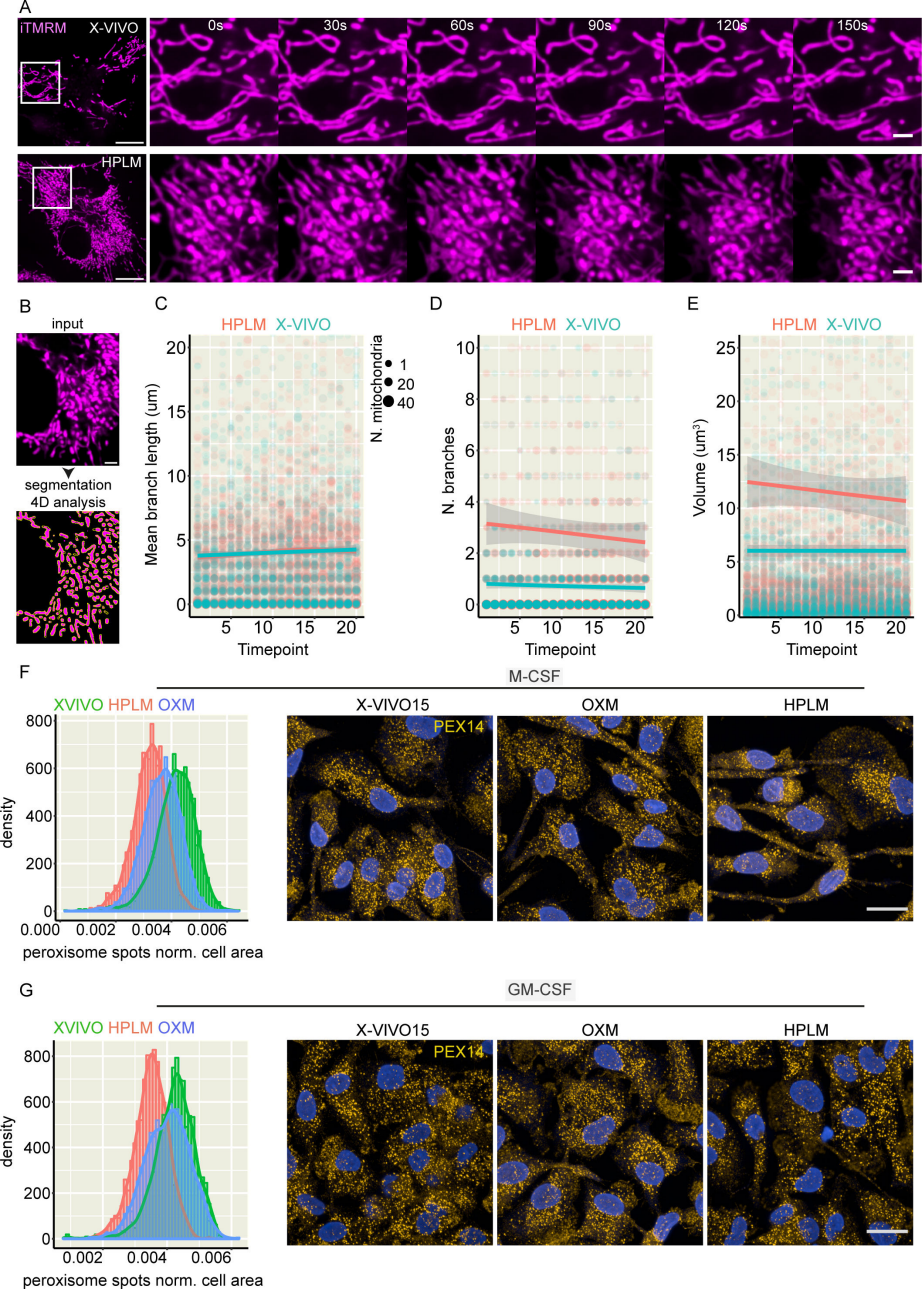
Cell culture media impacts on mitochondrial dynamics. (A) Live-cell super-resolution imaging evaluating mitochondrial dynamics in iPSDM differentiated with M-CSF and cultured using X-VIVO15 (top panel) or HPLM (bottom panel). Scale bar: 10 mm and 2 mm (zoom-in). (B) Schematic showing the 4D analysis sequence. (C–E) Dot plots show the quantification of mitochondrial mean branch length, number of branches, and volume over 20 time points (30 s timeframe) from one out of three independent experiments using iPSDM as described in as in panel A. (F, G) Histogram showing the quantification of PEX14-positive peroxisome spots per cell and representative images of iPSDM differentiated with M-CSF or GM-CSF, cultured in the indicated media and stained for PEX-14. *n* = 3 independent experiments. Scale bar: 20 mm

We next evaluated whether the changes in mitochondrial activity observed in HPLM-differentiated macrophages impact mitochondrial dynamics. A live-cell super-resolution and 3D z-stack analysis of the mitochondrial network (23) show that although the mean length and number of mitochondrial branches did not change over time, macrophages cultured in HPLM consistently exhibited a greater number of mitochondrial branches compared with X-VIVO 15-differentiated macrophages. This suggests that HPLM induces a more dynamic mitochondrial network in macrophages (Fig. 6A through D). Consistent with our high-content imaging analysis, the total mitochondrial volume was also greater in HPLM-differentiated macrophages (Fig. 6E). Together with LD and mitochondria, peroxisomes are central modulators of lipid metabolism that actively participate in metabolic processes such as fatty acid beta-oxidation and ether phospholipid synthesis (24). We quantified the peroxisome numbers per cell using single-cell high-content imaging (25) and found that X-VIVO 15-differentiated macrophages with either M-CSF or GM-CSF showed an increased number of peroxisomes per cell compared with iPSDM cultured in OXM or HPLM (Fig. 6F and G). Altogether, these data show that media composition is a main factor regulating macrophage metabolism-related organelle content, particularly lysosomes, LD, mitochondria, and peroxisomes with changes in metabolism.

### Lipid droplet content defines *M. tuberculosis* replication outcomes in human macrophages

**Fig 7:**
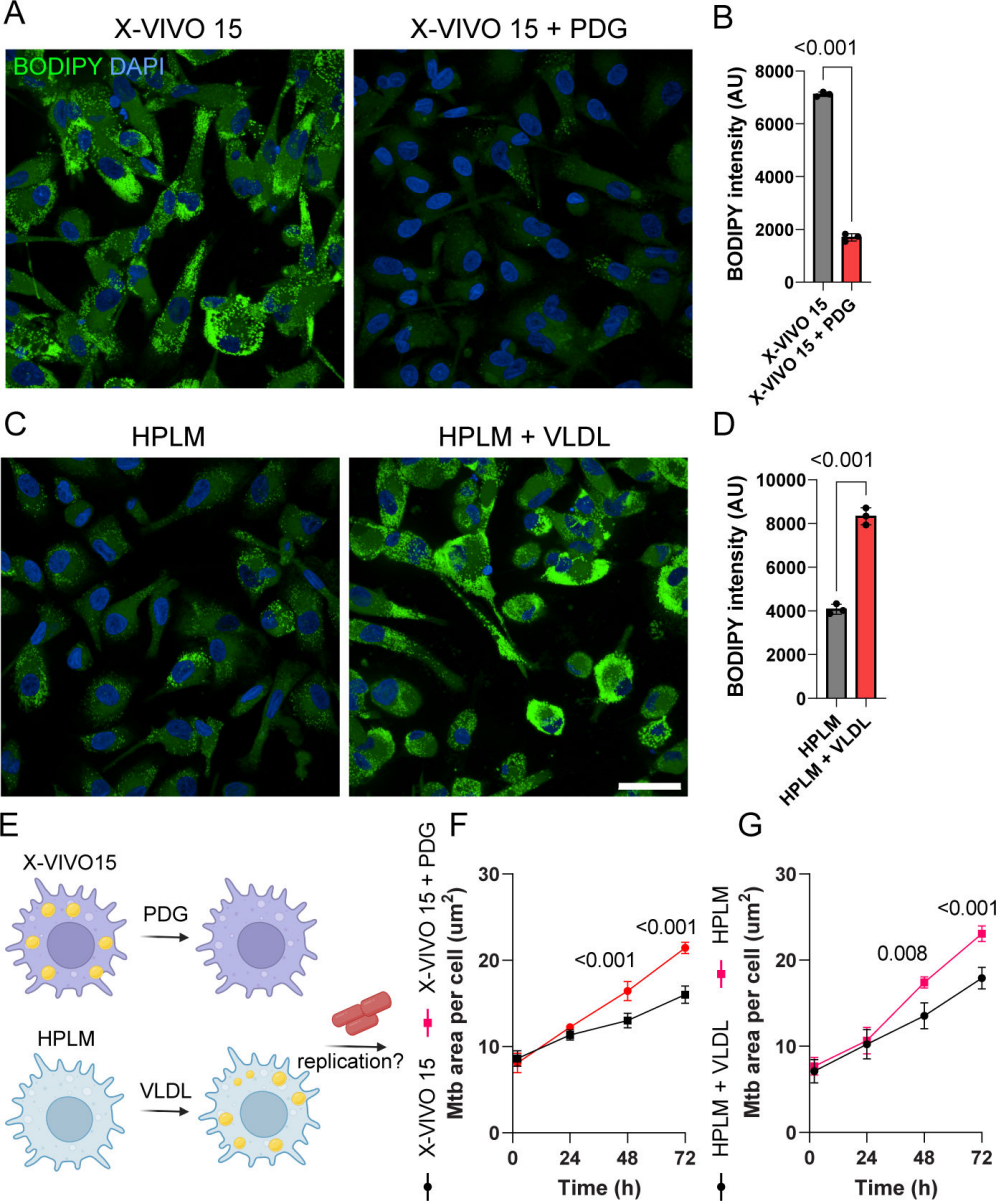
Culture media-induced lipid droplet content generates iPSDM more permissive or restrictive to *M. tuberculosis* replication. (A) Representative images of BODIPY-stained M-CSF differentiated iPSDM cultured in XVIVO-15 in the presence or absence of pradigastat (PDG). (B) Bar plots show BODIPY intensity quantification of iPSDM cultured in X-VIVO15 in the presence or absence of PDG. (C) Representative images of BODIPY-stained M-CSF differentiated iPSDM cultured in HPLM in the presence or absence of VLDL particles. (D) Bar plots show BODIPY intensity quantification of iPSDM cultured in HPLM in the presence or absence of VLDL particles. *n* = 3 independent experiments, *t*-test. (E) Scheme illustrating the experimental strategy to evaluate the impact of LD on Mtb replication. (F) Quantification of Mtb replication (bacteria area per cell) in iPSDM cultured in X-VIVO15 in the presence or absence of PDG. (G) Quantification of Mtb replication (bacteria area per cell) in iPSDM cultured in HPLM in the presence or absence of VLDL particles. *n* = 3 independent experiments, two-way ANOVA, Sidak’s multiple comparison test.

To validate whether the enrichment in LD content and lipid metabolism-associated pathways that we observed using non-physiological cell culture media has an impact on the macrophage host response, we pharmacologically modulated the LD content. Using the diacylglycerol acyltransferase 1 (DGAT1) inhibitor pradigastat (PDG) (18), we almost completely blocked LD production in X-VIVO 15-differentiated macrophages (Fig. 7A and B). In contrast, HPLM-differentiated macrophages, characterized by a small abundance of LD, significantly enhanced their LD abundance in the presence of triacylglycerol-enriched very low-density lipoproteins (VLDL) (26, 27) (Fig. 7A through C). Importantly, PDG-treated showed an increase in Mtb replication after 72 h of infection compared with X-VIVO 15-differentiated macrophages. On the other hand, HPLM-differentiated macrophages in the presence of VLDL particles presented a more restrictive environment for Mtb (Fig. 7E and F). Altogether, our data highlight the phenotypic impact of the cell culture media that closely reflects the metabolic profile of human plasma on organelle function and provides evidence that LD content is an intracellular determinant for Mtb replication and host control of infection.

## DISCUSSION

Conventional cell culture media, formulated decades ago, inadequately replicate the metabolic composition of human blood (7). Despite the advancing understanding of environmental influences on metabolism, these traditional media continue to serve as the standard for *in vitro* studies across diverse biological research disciplines (7). The emergence of physiologic media, along with other initiatives aimed at enhancing the modeling capabilities of cell culture (28), harbors significant promise for advancing our understanding of fundamental cellular processes (5, 29–31). This is particularly relevant in the context of the establishment of defined and robust culture conditions for iPSC and iPSC-differentiated cell types.

Here, we investigated how media composition influences human macrophage organelle content and activity in the context of host-pathogen responses to *M. tuberculosis* infection. We found profound changes at the transcriptional, metabolic, and organelle levels in macrophages cultured in traditional media compared with those cultured in media that closely reflect metabolite availability in human blood. iPSDM cultured in OXM or X-VIVO15 were characterized by enrichment in lipid metabolism transcriptional programs, reduced metabolic plasticity, and diminished lysosomal proteolytic activity and mitochondrial dynamics. The role of host lipids, and particularly LD, during mycobacterial infections has received significant attention in the context of tuberculosis (TB) pathogenesis, often yielding contrasting results that vary based on the cell type, infection conditions, and species studied (18, 26, 32–34). However, whether these differences might be influenced by or caused by the cell culture media employed has not been characterized. Our results suggest that the cell culture media employed could be a critical factor to consider when interpreting results in the context of LD in macrophages. In agreement with previous evidence showing a protective role for LD in the host defense to Mtb (18, 33) and other mycobacteria (26), we identified in iPSDM that increased LD content induced by OXM or X-VIVO15 culture media created a restrictive environment for Mtb replication in comparison to HPLM-differentiated iPSDM.

Traditionally, macrophage phenotypes and subtype classification have mainly been defined by distinct cell surface markers (35). However, our data challenge this paradigm, showing that macrophages with similar surface marker profiles can exhibit substantial variations in organelle content and metabolic activity. These differences significantly impact their effector functions, as demonstrated in their response to Mtb. Consequently, a more comprehensive analysis, including the quantification of organelles and metabolic characterization, would enhance our understanding of the full spectrum of macrophage states, from homeostasis to activation, revealing the nuanced differences that contribute to their functional heterogeneity.

Increasing evidence demonstrates that understanding organellar homeostasis is central to efforts aiming to identify new innate immune pathways (36). Our data, revealing the contrasting organelle and metabolic functions of human iPSDM cultured in different media, underscores the importance of adopting physiologic media as a standardized practice for *in vitro* functional studies. There is also a significant impact of medium composition on gene essentiality in human cells (29). Considering these findings, we anticipate that our study will pave the way for future investigations aimed at unraveling molecular mechanisms that are only discernible when employing a human plasma-like medium.

## MATERIALS AND METHODS

### Cells

#### iPSC and iPSDM culture

KOLF2 human iPSCs were sourced from Public Health England Culture Collections (catalog numbers 77650059 and 77650100, respectively) and maintained in Vitronectin XF (StemCell Technologies) coated plates with E8 medium (ThermoFisher Scientific). Cells were authenticated by short tandem repeats (STR) profiling upon receipt and were checked monthly for Mycoplasma contamination by PCR. Cells were passaged 1:6 once at 70% confluency using Versene (Gibco). Monocyte factories were set up following a previously reported protocol (van Wilgenburg et al., (1). Briefly, a single cell suspension of iPSCs was produced with TryplE (Gibco) at 37°C for 5 min and resuspended in E8 plus 10 µM Y-27632 (Stem Cell Technologies) and seeded into AggreWell 800 plates (StemCell Technologies) with 4 × 10^6^ cells/well and centrifuged at 100 g for 3 min. The forming embryonic bodies (EBs) were fed daily with two 50% medium changes with E8 supplemented with 50 ng/mL hBMP4 (Peprotech), 50 ng/mL hVEGF (Peprotech), and 20 ng/mL hSCF (Peprotech) for 3 days. On day 4, the EBs were harvested by flushing out of the well with gentle pipetting and filtered through an inverted 40 µm cell strainer. EBs were seeded at 100–150 EBs per T175 or 250–300 per T225 flask in factory medium consisting of OXM (4) supplemented with Glutamax (Gibco), 50 µM β-mercaptoethanol (Gibco), 100 ng/mL hM-CSF (Peprotech), and 25 ng/mL hIL-3 (Peprotech). These monocyte factories were fed weekly with factory medium for 5 weeks until plentiful monocytes were observed in the supernatant. Up to 50% of the supernatant was harvested weekly and factories were fed with 10–20 mL factory medium. The supernatant was centrifuged at 300 g for 5 min, and cells were resuspended in X-VIVO15 (2) (Lonza), OXM (4), or HPLM (A4899101) (3) supplemented with 100 ng/mL hM-CSF and plated at 4 × 10^6^ cells per 10 cm petri dish to differentiate over 7 days. HPLM medium was also supplemented with 10% dialyzed FBS (26400044, Thermo Fischer) as previously described (3). On day 4, a 50% medium change was performed. To detach cells, iPSDM plates were washed once with PBS and then incubated with EDTA for 15 min at 37°C and 5% CO_2_ before diluting 1:3 with PBS and gently scraping. Macrophages were centrifuged at 300 g and plated for experiments containing the respective media.

### Mtb infection

Mtb experiments were performed under BSL-3 conditions. Mtb H37Rv WT and Mtb H37Rv ΔRD1 were kindly provided by Prof. Douglas Young (The Francis Crick Institute, UK) and Dr Suzie Hingley Wilson (University of Surrey, UK). Fluorescent Mtb strains were generated as previously reported (37). E2Crimson Mtb was generated by transformation with pTEC19 (Addgene 30178, deposited by Prof. Lalita Ramakrishnan). Strains were verified by sequencing and tested for PDIM positivity by thin-layer chromatography of lipid extracts from Mtb cultures. Mtb strains were cultured in Middlebrook 7H9 supplemented with 0.2% glycerol, 0.05% Tween-80, and 10% albumin dextrose catalase (ADC). Mid-logarithmic-phase bacterial cultures (OD_600_ 0.5–1.0) were centrifuged at 2,000 × *g* for 5 min and washed twice in PBS. Pellets were then shaken vigorously for 1 min with 2.5–3.5 mm glass beads (VWR, 332124 G), and bacteria were resuspended in 10 mL macrophage culture medium before being centrifuged at 300 × *g* for 5 min to remove large clumps. The top 7 mL of bacterial suspension was taken; the OD_600_ was recorded, and the suspension was diluted appropriately for infection. To maintain consistency with the high-content organelle activity evaluations, HPLM medium was used to prepare the bacterial solution and was also used for the 2-hour uptake time point for all conditions. After 2 hours of uptake, extracellular bacteria were removed with two washes in PBS, and macrophages were incubated at 37°C and 5% CO_2_ in the corresponding media. At the required time post-infection, cells were collected or fixed in 4% paraformaldehyde (PFA). A target multiplicity of infection (MOI) of 1 was used for all the experiments, assuming OD_600_ of 1 is 1  ×  10^8^ bacteria mL^−1^.

### Seahorse-based metabolic flux analysis

iPSDM were seeded onto XF96 cell culture microplates (101085–004, Agilent Technologies) and assayed on a Seahorse XFe96 Analyzer (Agilent Technologies). OCR and extracellular acidification rates (ECAR) were measured in XF DMEM assay medium with pH adjusted to 7.4 (103680-100, Agilent Technologies) containing 10 mM glucose (103577-100, Agilent Technologies), 2 mM L-glutamine (103579-100, Agilent Technologies), and 1 mM sodium pyruvate (103578-100, Agilent Technologies). To investigate mitochondrial respiration and energetic phenotypes, a Seahorse XFp Cell Mito Stress Test kit (103010-100, Agilent Technologies) was used. The injection strategy was as follows: first, oligomycin (1 mM at the final concentration); second, carbonyl cyanide 4-(trifluoromethoxy) phenylhydrazone (FCCP) (1 mM at the final concentration); and third, rotenone and antimycin A (0.5 mM at the final concentration).

After finishing the assay, cells were fixed with PFA 4% for 15 min. After that, nuclei were stained with 4′,6-diamidino-2-phenylindole (DAPI) and imaged using an EVOS microscope (Thermo Fischer). The Analyze Particles command from ImageJ was used for nuclei quantification, and when required, cell number normalization was performed. WAVE software, version 2.6.1 (Agilent Technologies), was used for data analysis.

### Flow cytometry

Cells were collected and incubated in PBS plus 0.1% bovine serum albumin (BSA) (9998S; Cell Signaling Technologies) and 5 µL Fc block per million cells for 20 min. In total, 50 µL of cells were then incubated with 50 µL antibody cocktail diluted in PBS and 0.1% BSA for 20 min on ice in the dark. Cells were washed in 2 mL PBS and fixed in 2% PFA (15710; Electron Microscopy Sciences) diluted in PBS prior to analysis. Cells were analyzed on an LSRII flow cytometer. Antibodies were purchased from BD Biosciences Antibody (CD14-Alexa488, 562689; CD119-PE, 558934; CD86-BV421, 562433; CD11b-BV421, 562632; CD163-FITC, 563697; CD169-PE, 565248; CD206-APC, 561763; CD16-Alexa647, 557710; Alexa488 isotype, 557703; Alexa647 isotype, 57714; PE isotype, 12–4015-82; BV421 isotype, 562438; CD16-Alexa647, 557710; Alexa488 isotype, 557703). Flow cytometry data were analyzed and plotted in FlowJo (BD Biosciences).

### Imaging

#### High-content live-cell imaging

iPSDM (30,000) were seeded into a 96-well flat-bottom PhenoPlate (PerkinElmer). After overnight incubation, iPSDM were imaged after incubation with the indicated probes or infected with *M. tuberculosis* as described above. The plate was sealed with parafilm and placed in a pre-heated (37°C) Opera Phenix microscope (PerkinElmer) with 5% CO_2_. Water-immersion lenses (40× or 60×) were used, and capture settings were as follows: BODIPY 493/503 (D3922, Thermo Fischer) was excited with the 488 nm laser at 5% power with 100 ms exposure, Image-iT TMRM (I34361, Thermo Fischer) was excited with the 561 nm laser at 10% power with 100 ms exposure, and iABP probe and Mtb E2crimson were excited with the 640 nm laser at 10% power with 100 ms exposure. DAPI was excited with the 405 nm laser at 20% power with 100 ms exposure. At least 20 fields per well were imaged in all the experiments. Images were acquired at 1,020 × 1,020 pixels using Harmony 4.9 high-content imaging and analysis software (PerkinElmer).

#### Organelle staining in live cells

After the indicated treatments, cells were washed once with PBS and incubated at 37°C and 5% CO_2_ with Image-iT TMRM Reagent (1:1,000 solution, 20 min); BODIPY 493/503 (1 mg/L solution, 30 min); iABP probe (1 mm solution, 30 min) for the evaluation of mitochondrial membrane potential, LD content and lysosomal proteolytic activity, respectively. To avoid differences in probe uptake dependent on the media, the probes were always resuspended in HPLM, and all the measurements were carried out in the same media. Nuclear staining was done using 300 nM DAPI (Life Technologies, D3571), or NucBlue Live ReadyProbes Reagent (Hoechst 33342) and incubated simultaneously with the mitochondrial probe. After that, cells were gently washed with PBS and replaced with fresh HPLM media before imaging acquisition. Fluorescence intensities were quantified after single-mitochondrion and single-cell segmentation as described later (imaging analysis).

#### PEX14 staining

After the indicated treatments, iPSDM were washed once with PBS and fixed with 4% methanol-free PFA in PBS for 15 min. After three washes with PBS, cells were permeabilized using a 0.5% Triton X-100 (Sigma)/PBS solution for 10 min. Cells were then immunostained using anti-PEX14 antibody (1:400 dilution) (ab183885, Abcam). After 1 h, cells were washed twice with PBS and incubated with a 1:700 solution of anti-rabbit Alexa Fluor 546 antibody (A-11035, Thermo Fischer). Antibodies were diluted in PBS containing 5% FBS and incubated for 1 h at room temperature. iPSDM were then washed with PBS, and DAPI was added for nuclear detection and cell segmentation (imaging analysis).

#### Super-resolution live-cell imaging

iPSDM incubated with Image-iT TMRM were imaged on a VT-iSIM super-resolution imaging system (Visitech International), using an Olympus IX83 microscope, 100 ×/1.5 Apochromat objective (Olympus), ASI motorized stage with piezo Z, and 2 × Prime BSI Express scientific CMOS cameras (Teledyne Photometrics). Cells were always in the stage incubator at 37°C and 5% CO_2_. mCherry imaging was done using the 560 nm laser excitation and ET600/50 m emission filters (Chroma). Z-stacks (100 nm z-step) were acquired at the intervals indicated in the figure legends. The microscope was controlled with CellSens software (Olympus).

### Imaging analysis

#### Organelle segmentation and analysis

Image-iT TMRM (mitochondrial) fluorescence intensity was quantified at the single mitochondrial level and analyzed as the mean mitochondrial fluorescence intensity per cell. Single-mitochondrial and single-cell segmentation were done in Harmony 4.9 software using SER texture building block and nuclear staining, respectively. BODIPY (lipid droplets), iABP (lysosomes), and PEX-14 positive puncta (peroxisomes) fluorescence intensity was quantified at the single-segmented object (organelle) level and analyzed as the mean object fluorescence intensity per cell. Single-organelle segmentation was done in Harmony 4.9 software using the Find Spots module. Cells were single-cell segmented based on nuclear staining and using the Find Nuclei and Morphology Properties modules in Harmony 4.9. Mtb-infected iPSDM were identified after segmentation of bacteria area per cell, as indicated below (Mtb replication analysis).

#### Live-cell super-resolution analysis

Mitochondrial morphology and dynamics were evaluated using the Mitochondria Analyzer plugin for ImageJ/Fiji keeping the default settings for 4D data sets as previously described (23).

### RNA processing

iPSDM (10^6^) were seeded in 6-well plates and cultured in the respective media. RNA extraction was done by incubating samples with 1 mL TRIzol (15596026, Thermo Fischer). Samples were stored at −80°C, and RNA extraction was done using a Direct-zol RNA Miniprep Plus (R2070, Zymo Research).

### RNA-sequencing and analysis

Samples were normalized at 200 ng total RNA and after polyA selection; libraries were prepared using NEB Ultra II Directional RNA Library Prep Kit following the manufacturer’s instructions. Libraries were pooled and sequenced on Illumina NovaSeq 6000 with 100 bp paired-end reads. The raw RNA-Seq fastq files were adaptor and quality-trimmed using Trimmomatic (v0.40) before being aligned to human genome GRCh38 build 88 using STAR (v2.7.4a). Gene counting was performed using RNA-Seq by Expectation-Maximization(RSEM) (v1.3.1), and the raw gene counts were normalized using DESeq2 (v1.42.0) where differential expression between the two groups was calculated using Wald statistics and adjusted for multiple testing, false discovery rate (FDR) = 0.05. Venn diagrams were created using the ggvenn package (v0.1.10) in R with up- or downregulated DEGs (filtered by fold change > 1.2 and adjusted *P*-value < 0.05 when comparing GM-CSF with M-CSF) in each culture medium. DEGs that are common across all three media with the same directionality were extracted for pathway analysis using Reactome (v88) to identify overrepresented pathways.

### Statistical analysis

Statistical analysis was performed using GraphPad Prism 10 software or R Studio 2023.03.0 (R version 4.2.2). High-content imaging analysis and mean values were obtained using R 4.2.2 or Harmony 4.9 software. The number of biological replicates, the statistical analysis performed, and post-hoc tests used are mentioned in the figure legends. The statistical significance of data is denoted on graphs by the corresponding *P*-values or with asterisks, where * = *P* < .05, ** = *P* < .01, *** = *P* < .001, or ns = not significant. Bar plots were plotted in GraphPad Prism software. Density and dot plots were plotted using R 4.2.2. Schemes were created with BioRender.com.

## Data Availability

RNA-Seq data were deposited into the Gene Expression Omnibus database under accession number GSE255475.

## AFTER EPUB

*[This article was published on 10 July 2024 with errors in Fig. 7. The figure was corrected in the current version, posted on 12 July 2024.]*

## References

[1] van Wilgenburg B, Browne C, Vowles J, Cowley SA. 2013. Efficient, long term production of monocyte-derived macrophages from human pluripotent stem cells under partly-defined and fully-defined conditions. PLoS One 8:e71098. doi:10.1371/journal.pone.0071098 23951090 PMC3741356

[2] Bernard EM, Fearns A, Bussi C, Santucci P, Peddie CJ, Lai RJ, Collinson LM, Gutierrez MG. 2020. M. tuberculosis infection of human iPSC-derived macrophages reveals complex membrane dynamics during xenophagy evasion. J Cell Sci 134:jcs252973. doi:10.1242/jcs.252973 32938685 PMC7710011

[3] Cantor JR, Abu-Remaileh M, Kanarek N, Freinkman E, Gao X, Louissaint A Jr, Lewis CA, Sabatini DM. 2017. Physiologic medium rewires cellular metabolism and reveals uric acid as an endogenous inhibitor of UMP synthase. Cell 169:258–272. doi:10.1016/j.cell.2017.03.023 28388410 PMC5421364

[4] Vaughan-Jackson A, Stodolak S, Ebrahimi KH, Browne C, Reardon PK, Pires E, Gilbert-Jaramillo J, Cowley SA, James WS. 2021. Differentiation of human induced pluripotent stem cells to authentic macrophages using a defined, serum-free, open-source medium. Stem Cell Reports 16:1735–1748. doi:10.1016/j.stemcr.2021.05.018 34171284 PMC8282471

[5] Vande Voorde J, Ackermann T, Pfetzer N, Sumpton D, Mackay G, Kalna G, Nixon C, Blyth K, Gottlieb E, Tardito S. 2019. Improving the metabolic fidelity of cancer models with a physiological cell culture medium. Sci Adv 5:eaau7314. doi:10.1126/sciadv.aau7314 30613774 PMC6314821

[6] Psychogios N, Hau DD, Peng J, Guo AC, Mandal R, Bouatra S, Sinelnikov I, Krishnamurthy R, Eisner R, Gautam B, Young N, Xia J, Knox C, Dong E, Huang P, Hollander Z, Pedersen TL, Smith SR, Bamforth F, Greiner R, McManus B, Newman JW, Goodfriend T, Wishart DS. 2011. The human serum metabolome. PLoS One 6:e16957. doi:10.1371/journal.pone.0016957 21359215 PMC3040193

[7] Cantor JR. 2019. The rise of physiologic media. Trends Cell Biol 29:854–861. doi:10.1016/j.tcb.2019.08.009 31623927 PMC7001851

[8] Golikov MV, Valuev-Elliston VT, Smirnova OA, Ivanov AV. 2022. Physiological media in studies of cell metabolism. Mol Biol 56:629–637. doi:10.1134/S0026893322050077 36217338 PMC9534458

[9] Fischer M. 2023. Physiological media advance cell culture experiments. Trends Biochem Sci 48:103–105. doi:10.1016/j.tibs.2022.08.007 36114088

[10] Ushach I, Zlotnik A. 2016. Biological role of granulocyte macrophage colony-stimulating factor (GM-CSF) and macrophage colony-stimulating factor (M-CSF) on cells of the myeloid lineage. J Leukoc Biol 100:481–489. doi:10.1189/jlb.3RU0316-144R 27354413 PMC4982611

[11] Hamidzadeh K, Belew AT, El-Sayed NM, Mosser DM. 2020. The transition of M-CSF-derived human macrophages to a growth-promoting phenotype. Blood Adv 4:5460–5472. doi:10.1182/bloodadvances.2020002683 33166408 PMC7656919

[12] Lacey DC, Achuthan A, Fleetwood AJ, Dinh H, Roiniotis J, Scholz GM, Chang MW, Beckman SK, Cook AD, Hamilton JA. 2012. Defining GM-CSF- and macrophage-CSF-dependent macrophage responses by in vitro models. J Immunol 188:5752–5765. doi:10.4049/jimmunol.1103426 22547697

[13] Locati M, Curtale G, Mantovani A. 2020. Diversity, mechanisms, and significance of macrophage plasticity. Annu Rev Pathol 15:123–147. doi:10.1146/annurev-pathmechdis-012418-012718 31530089 PMC7176483

[14] Bussi C, Heunis T, Pellegrino E, Bernard EM, Bah N, Dos Santos MS, Santucci P, Aylan B, Rodgers A, Fearns A, Mitschke J, Moore C, MacRae JI, Greco M, Reinheckel T, Trost M, Gutierrez MG. 2022. Lysosomal damage drives mitochondrial proteome remodelling and reprograms macrophage immunometabolism. Nat Commun 13:7338. doi:10.1038/s41467-022-34632-8 36443305 PMC9705561

[15] Na YR, Gu GJ, Jung D, Kim YW, Na J, Woo JS, Cho JY, Youn H, Seok SH. 2016. GM-CSF induces inflammatory macrophages by regulating glycolysis and lipid metabolism. J Immunol 197:4101–4109. doi:10.4049/jimmunol.1600745 27742831

[16] Wessendarp M, Watanabe-Chailland M, Liu S, Stankiewicz T, Ma Y, Kasam RK, Shima K, Chalk C, Carey B, Rosendale L-R, Dominique Filippi M, Arumugam P. 2022. Role of GM-CSF in regulating metabolism and mitochondrial functions critical to macrophage proliferation. Mitochondrion 62:85–101. doi:10.1016/j.mito.2021.10.009 34740864 PMC9573767

[17] Saha S, Shalova IN, Biswas SK. 2017. Metabolic regulation of macrophage phenotype and function. Immunol Rev 280:102–111. doi:10.1111/imr.12603 29027220

[18] Greenwood DJ, Dos Santos MS, Huang S, Russell MRG, Collinson LM, MacRae JI, West A, Jiang H, Gutierrez MG. 2019. Subcellular antibiotic visualization reveals a dynamic drug reservoir in infected macrophages. Science 364:1279–1282. doi:10.1126/science.aat9689 31249058 PMC7012645

[19] Evers BM, Rodriguez-Navas C, Tesla RJ, Prange-Kiel J, Wasser CR, Yoo KS, McDonald J, Cenik B, Ravenscroft TA, Plattner F, Rademakers R, Yu G, White CL, Herz J. 2017. Lipidomic and transcriptomic basis of lysosomal dysfunction in progranulin deficiency. Cell Rep 20:2565–2574. doi:10.1016/j.celrep.2017.08.056 28903038 PMC5757843

[20] Kaushik S, Cuervo AM. 2015. Degradation of lipid droplet-associated proteins by chaperone-mediated autophagy facilitates lipolysis. Nat Cell Biol 17:759–770. doi:10.1038/ncb3166 25961502 PMC4449813

[21] Verdoes M, Oresic Bender K, Segal E, van der Linden WA, Syed S, Withana NP, Sanman LE, Bogyo M. 2013. Improved quenched fluorescent probe for imaging of cysteine cathepsin activity. J Am Chem Soc 135:14726–14730. doi:10.1021/ja4056068 23971698 PMC3826460

[22] Rial E, Rodríguez-Sánchez L, Gallardo-Vara E, Zaragoza P, Moyano E, González-Barroso MM. 2010. Lipotoxicity, fatty acid uncoupling and mitochondrial carrier function. Biochim Biophys Acta 1797:800–806. doi:10.1016/j.bbabio.2010.04.001 20388489

[23] Chaudhry A, Shi R, Luciani DS. 2020. A pipeline for multidimensional confocal analysis of mitochondrial morphology, function, and dynamics in pancreatic beta-cells. Am J Physiol Endocrinol Metab 318:E87–E101. doi:10.1152/ajpendo.00457.2019 31846372 PMC7052579

[24] Lodhi IJ, Semenkovich CF. 2014. Peroxisomes: a nexus for lipid metabolism and cellular signaling. Cell Metab 19:380–392. doi:10.1016/j.cmet.2014.01.002 24508507 PMC3951609

[25] Pellegrino E, Aylan B, Bussi C, Fearns A, Bernard EM, Athanasiadi N, Santucci P, Botella L, Gutierrez MG. 2023. Peroxisomal ROS control cytosolic Mycobacterium tuberculosis replication in human macrophages. J Cell Biol 222:e202303066. doi:10.1083/jcb.202303066 37737955 PMC10515436

[26] Caire-Brändli I, Papadopoulos A, Malaga W, Marais D, Canaan S, Thilo L, de Chastellier C. 2014. Reversible lipid accumulation and associated division arrest of Mycobacterium avium in lipoprotein-induced foamy macrophages may resemble key events during latency and reactivation of tuberculosis. Infect Immun 82:476–490. doi:10.1128/IAI.01196-13 24478064 PMC3911402

[27] den Hartigh LJ, Connolly-Rohrbach JE, Fore S, Huser TR, Rutledge JC. 2010. Fatty acids from very low-density lipoprotein lipolysis products induce lipid droplet accumulation in human monocytes. J Immunol 184:3927–3936. doi:10.4049/jimmunol.0903475 20208007 PMC2843797

[28] Kyriakopoulou K, Koutsakis C, Piperigkou Z, Karamanos NK. 2023. Recreating the extracellular matrix: novel 3d cell culture platforms in cancer research. FEBS J 290:5238–5247. doi:10.1111/febs.16778 36929947

[29] Rossiter NJ, Huggler KS, Adelmann CH, Keys HR, Soens RW, Sabatini DM, Cantor JR. 2021. CRISPR screens in physiologic medium reveal conditionally essential genes in human cells. Cell Metab 33:1248–1263. doi:10.1016/j.cmet.2021.02.005 33651980 PMC8172426

[30] Pellegrino E, Gutierrez MG. 2021. Human stem cell-based models for studying host-pathogen interactions. Cell Microbiol 23:e13335. doi:10.1111/cmi.13335 33792137

[31] Avellino G, Deshmukh R, Rogers SN, Charnock-Jones DS, Smith GCS, Tardito S, Aye ILMH. 2023. Physiologically relevant culture medium plasmax improves human placental trophoblast stem cell function. Am J Physiol Cell Physiol 324:C878–C885. doi:10.1152/ajpcell.00581.2022 36878843 PMC10069969

[32] Barisch C, Soldati T. 2017. Breaking fat! How mycobacteria and other intracellular pathogens manipulate host lipid droplets. Biochimie 141:54–61. doi:10.1016/j.biochi.2017.06.001 28587792

[33] Knight M, Braverman J, Asfaha K, Gronert K, Stanley S. 2018. Lipid droplet formation in Mycobacterium tuberculosis infected macrophages requires IFN-gamma/HIF-1alpha signaling and supports host defense. PLoS Pathog 14:e1006874. doi:10.1371/journal.ppat.1006874 29370315 PMC5800697

[34] Kalam H, Chou C-H, Kadoki M, Graham DB, Deguine J, Hung DT, Xavier RJ. 2023. Identification of host regulators of Mycobacterium tuberculosis phenotypes uncovers a role for the MMGT1-GPR156 lipid droplet axis in persistence. Cell Host Microbe 31:978–992. doi:10.1016/j.chom.2023.05.009 37269834 PMC10373099

[35] Murray PJ. 2017. Macrophage polarization. Annu Rev Physiol 79:541–566. doi:10.1146/annurev-physiol-022516-034339 27813830

[36] Harapas CR, Idiiatullina E, Al-Azab M, Hrovat-Schaale K, Reygaerts T, Steiner A, Laohamonthonkul P, Davidson S, Yu C-H, Booty L, Masters SL. 2022. Organellar homeostasis and innate immune sensing. Nat Rev Immunol 22:535–549. doi:10.1038/s41577-022-00682-8 35197578

[37] Lerner TR, de Souza Carvalho-Wodarz C, Repnik U, Russell MRG, Borel S, Diedrich CR, Rohde M, Wainwright H, Collinson LM, Wilkinson RJ, Griffiths G, Gutierrez MG. 2016. Lymphatic endothelial cells are a replicative niche for Mycobacterium tuberculosis. J Clin Invest 126:1093–1108. doi:10.1172/JCI83379 26901813 PMC4767353

